# HIV-1 Clade D Is Associated with Increased Rates of CD4 Decline in a Kenyan Cohort

**DOI:** 10.1371/journal.pone.0049797

**Published:** 2012-11-21

**Authors:** Lyle R. McKinnon, Nico J. Nagelkerke, Rupert Kaul, Souradet Y. Shaw, Rupert Capina, Ma Luo, Anthony Kariri, Winnie Apidi, Makobu Kimani, Charles Wachihi, Walter Jaoko, A. Omu Anzala, Joshua Kimani, T. Blake Ball, Francis A. Plummer

**Affiliations:** 1 Department of Medicine, University of Toronto, Toronto, Canada; 2 Department of Medical Microbiology, University of Nairobi, Nairobi, Kenya; 3 Department of Medical Microbiology and 9 Immunology, University of Manitoba, Winnipeg, Canada; 4 Department of Public Health, Erasmus Medical Center, Rotterdam, The Netherlands; 5 Community Medicine, United Arab Emirates University, Al Ain, United Arab Emirates; 6 Centre for Global Public Health, University of Manitoba, Winnipeg, Canada; 7 National Microbiology Lab, Public Health Agency of Canada, Winnipeg, Canada; 8 Kenyan AIDS Vaccine Initiative, Nairobi, Kenya; 10 Laboratory of HIV Immunology, Public Health Agency of Canada, Winnipeg, Manitoba, Canada; Johns Hopkins University Bloomberg School of Public Health, United States of America

## Abstract

HIV-1 is grouped phylogenetically into clades, which may impact rates of HIV-1 disease progression. Clade D infection in particular has been shown to be more pathogenic. Here we confirm in a Nairobi-based prospective female sex worker cohort (1985–2004) that Clade D (n = 54) is associated with a more rapid CD4 decline than clade A1 (n = 150, 20.6% vs 13.4% decline per year, 1.53-fold increase, p = 0.015). This was independent of “protective” HLA and country of origin (p = 0.053), which in turn were also independent predictors of the rate of CD4 decline (p = 0.026 and 0.005, respectively). These data confirm that clade D is more pathogenic than clade A1. The precise reason for this difference is currently unclear, and requires further study. This is first study to demonstrate difference in HIV-1 disease progression between clades while controlling for protective HLA alleles.

## Introduction

HIV infection is characterized by a prolonged host-pathogen interaction that, in the absence of antiretroviral therapy, eventually leads to CD4 decline and AIDS. The rate of HIV disease progression is heterogeneous and driven by many factors [1]. A better understanding of the host, viral, and environmental factors that contribute to this heterogeneity would improve our understanding of HIV pathogenesis and therapeutic strategies. HIV exhibits extreme genetic diversity, in some cases differing by more than 30% amino acid homology in the Env protein. The HIV-1 main group (M), which accounts for most infections globally, has been classified into numerous clades (subtypes) and circulating recombinant forms (CRFs), with distinct geographical distributions [2]. In sub-Saharan Africa, where the pandemic originated [3], most countries have multiple clades and/or CRFs in circulation.

Several previous studies have assessed the impact of HIV subtype on the rate of disease progression. An early study in Kenya showed that clade C was associated with faster progression, higher viral loads, and lower CD4 counts than clades A1 or D [4]. Another study in Senegal demonstrated that infection by clades other than A1 was associated with an increased risk of disease progression during the period of follow-up [5]. Recent studies in Kenya, Tanzania, and Uganda suggest that clade D is associated with more rapid disease progression than clade A, ranging from approximately 1.3 to >6 times faster [6], [7], [8], [9]. It is unclear what immunological or virological mechanism(s) underlie these differences [10]. One report suggested that clade D was associated with CXCR4-tropism [11], which has previously been associated with faster progression to AIDS [12]. Therefore, while differences in progression between clades have been observed previously, it remains uncertain whether these are due to differences in viral replication. We sought to confirm the differences in CD4 decline between clades A1 and D at different stages of HIV-1 disease progression, and explore whether these were associated with differences in HIV viral loads *in vivo*.

## Methods

### Cohort Description

We enrolled participants from a large prospective female sex worker cohort in Nairobi, Kenya. All participants provided informed written consent, and Institutional Review Boards at Kenyatta National Hospital and University of Manitoba approved the study. HIV-1 clade was determined cross-sectionally by HIV-1 p24 proviral DNA sequencing, as described (n = 377) [13]. The region sequenced corresponds to HXB2 positions 1,508–2,198. Only clade A1 and D-infected participants were included; >90% of the cohort was infected by these clades. Class I HLA haplotypes were determined by DNA sequencing, as previously described [13]. HLA alleles were considered protective based on their ability to slow disease progression consistently in prior studies; protective alleles included HLA-B*1302, B*27, B*5101, B*5701/3, and B*8101 [14]. Participant characteristics and details of follow-up of those infected by clades A1 and D in the present study are shown in Table 1. CD4 counts were measured biannually in this cohort from 1990 onwards using Becton Dickinson Tritest reagents. The majority of the cohort (>80%) was HIV seropositive at enrollment. All participants were ART-naive for the period of follow-up in this study. Viral loads are not standard of care in this cohort and were measured, where available, using Roche bDNA viral load assay v. 3.0.

**Table 1:** Baseline characteristics of the study cohort.

Variable	Clade A1 (n = 150)	Clade D (n = 54)	P value
Mean age, years (median, IQR)	35 (30–39)	32 (27–38)	0.32
Kenyan	25.5%	14.8%	0.13
Follow-up, days (median, IQR)	2,438 (1,309–3,824)	2,057 (972–3,230)	0.159
CD4 count at baseline (median, IQR)	576 (453–740)	516 (413–689)	0.133
No. of CD4 counts (median, IQR)	11 (6–16)	8.5 (4–15)	0.077
Protective HLA	29.1%	18.9%	0.20
% with VL data	48.7%	35.2%	0.11
Average log_10_ copies/ml (VL; median, IQR)	3.74 (3.02–4.48)	3.95 (3.53–4.91)	0.186

### Statistical Approach

Chi-square and Mann-Whitney tests were used to compare participant characteristics, where appropriate. Slopes of CD4 decline were analyzed using unstructured linear mixed models with random intercept and slope. In order to “linearize” rates of decline, CD4 counts were transformed using the natural log (lnCD4). Natural log-derived parameters are more easily interpreted than square or cube roots, and previous reports suggest these measures behave similar, in terms of linearization of rates of decline, over the ranges studied [15]. In the most basic model, decline of lnCD4 was a dependent variable, with follow-up and the interaction between clade and time as outcome variables. Potential confounders were then added as additional co-variables including protective HLA (interacting with time), and country of origin (interacting with time).

## Results

### Participant Characteristics

Of the 377 participants with HIV-1 sequence data, 204 were included (54%) in this analysis on the basis of having a CD4>350 at baseline, as has been reported elsewhere [16]. No other exclusion criteria were used. Apart from shorter duration of follow-up and lower CD4 count (by definition), there were no differences between excluded individuals compared to those included in the analysis (not shown). Clade A1 was approximately three times more common than clade D. Baseline and follow-up data for study participants are shown in Table 1. Although there were no statistically significant differences, clade A1-infected participants tended to be older (median 35 vs. 32, p = 0.32), more likely to be Kenyan (25.5 vs. 14.8%, p = 0.13), and have at least one protective class I HLA allele (29.1 vs. 18.9%, p = 0.20). Other participants were from Tanzania (65.5%) and Uganda (11.8%). Clade A1 participants tended to have longer follow-up (median 2,438 vs. 2,057 days, p = 0.159) and more CD4 measurements (median 11 vs. 8.5, p = 0.077). Many of these are consistent with the hypothesized longer survival time for clade A1 compared to D-infected individuals. Since VL is not standard of care, these were only available for a random selection of clade A1 (48.7%) and D (35.2%) participants.

### Impact of Clade and Co-variates on Slope of CD4 Decline

**Figure 1:**
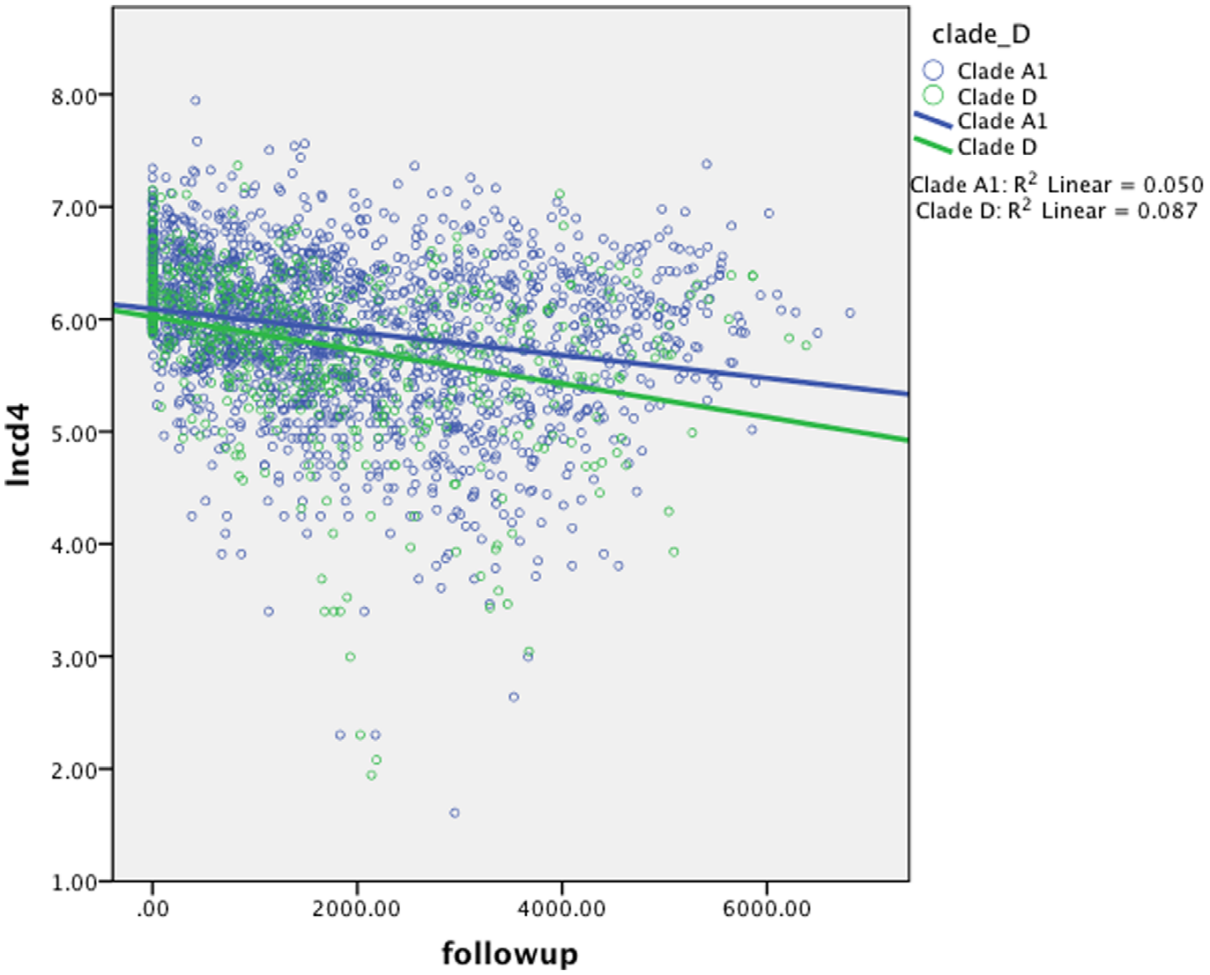
Scatter plot showing slopes of decline of natural log CD4 (lnCD4, y-axis) over time (x-axis). Dots indicate individual CD4 measures, while lines indicate mean slope of decline for participants infected by Clades A1 (blue) and clade D (green). These were analyzed using linear mixed models (Table 2).

Because time of infection is left-censored for the majority of the cohort, survival analyses to determine the impact of clade on time to CD4 decline below a fixed threshold (e.g. 350) were not feasible. Therefore we used linear mixed models with random effects (slope and intercept) to determine the impact of clade on CD4 decline during prospective follow-up. The natural log of CD4 (lnCD4) was the dependent variable in all models; this measure declines linearly and is assumed to be independent of time of infection. In the most basic model (Table 2, Model 1), including time and the interaction between clade D and time, clade D was associated with a more rapid CD4 decline (p = 0.015, annual estimate = 0.061, 95% CI 0.012, 0.110). While clade D-infected individuals experienced an annual CD4 decline of 20.6%, this decline was 13.4% for clade A1, translating to a 1.53-fold more rapid CD4 decline for clade D than A1 (Figure 1). While CD4 cells declined by an average of 39.7 cells/year for clade A1, clade D infected participant CD4 counts declined by 50.6 cells/year. In an exploratory analysis including only participants with a CD4>500 at baseline, the impact of clade D on CD4 decline was also observed (not shown).

**Table 2:** Results from linear mixed models examining factors associated with CD4 decline.

Variable	Model 1		Model 2	
	Estimate (95% CI)	P value	Estimate (95% CI)	P value
Follow Up	−0.187 (−0.230, −0.144)	<0.001	−0.084 (−0.156, −0.012)	0.022
Clade A1*follow-up	0.061 (0.012, 0.110)	0.015	0.048 (−0.001, 0.097)	0.053
No Protective HLA* follow-up	−	−	−0.054 (−0.102, −0.007)	0.026
Kenyan*follow-up	−	−	0.070 (−0.119, −0.021)	0.005

Next we controlled for various potential confounders (Table 2, Model 2). Age or duration of sex work had no impact on the association between clade and CD4 decline. Baseline CD4 was associated with time but also did not affect the clade association (not shown). No associations between baseline CD4 and clade, protective HLA, or country of origin were observed, and these were omitted from further models (not shown). In a multivariate model (Model 2), both protective HLA alleles and country of origin were independently associated with rate of CD4 decline (No protective HLA, p = 0.026, Estimate −0.054, 95% CI −0.102, −0.007; Non-Kenyan, p = 0.005, Estimate −0.070, 95% CI −0.119, −0.121), and these decreased the effect observed for clade (p = 0.053, Estimate 0.048, 95% CI −0.001, 0.097). Lastly, we added average VL as a co-variate to determine its impact on rates of CD4 decline. This was a sub-analysis that included VL data where available (92/204 participants with baseline CD4>350). While follow-up and country of origin remain significant in this model (p = 0.019 and p = 0.021, respectively), clade and protective HLA were no longer associated with rates of CD4 decline (not shown).

## Discussion

These data support earlier findings that the rate of CD4 decline among individuals infected with clade D is more rapid, by approximately 50%, than those infected with clade A1. Despite the consistency of this observation, the reason(s) that underlie it remain unclear. One hypothesis is that clade D is associated with a higher VL, perhaps as a result of increased replicative fitness. Other hypotheses include that clade D viruses have a differential effect on other factors that impact HIV-1 disease progression, such as cellular tropism or immune activation. Resolution of these differences could provide important data for better understanding the heterogeneity of HIV-1 disease progression.

Our data suggest there is a slight (0.2 log_10_ copies/ml) increase in VL associated with clade D that was not statistically significant. While clade was a reasonable predictor of the slope of CD4 decline in models that include several relevant co-variates, the addition of VL nullified this association (while follow-up remained significant). It is important to note a major limitation of our study is that VL could only be included as a sub-analysis, and the time point of infection for VL remains unknown. Therefore it does not appear VL is a major determinant of the difference in rates of disease progression between clades. Another possible future direction would be to compare reservoir size between clades A1 and D by measuring cell-associated VL.

Our study is the first to compare differences in HIV-1 disease progression between clades while controlling for protective HLA alleles. These data suggest that both clade and HLA can contribute to rates of disease progression. HLA differences between populations could be an important confounder in studies of HIV pathogenesis. In addition, country of origin was a strong, independent predictor of the rate of CD4 decline in all models. The reasons for this are still not clear and suggest an impact of other, unmeasured, confounders.

Previous studies of HIV-1 clade and disease have employed several different approaches. While the methods to determine clade are likely all valid, the HIV-1 region under study could be important. In our study, clade was determined by sequencing and phylogenetics of the p24 protein of HIV-1. Therefore, one potential limitation is that recombinants may be missed due to misclassification bias. On the contrary, our data suggest that clade of p24 might be an important predictor of both VL and rates of CD4 decline. Studies of CTL escape suggest p24 has many conserved residues that are likely structurally important for virion function [17], [18]. Study of the p24 differences between clades therefore might be an important avenue for future study.

In summary, these data add to mounting evidence that clade D is more pathogenic than clade A1. Some studies suggest clade A1 is associated with increased transmission [19], [20], possibly because clade D viruses tend to be X4-tropic, and therefore do not readily establish infection [21]. The above data suggest that any difference in pathogenecity between clades will not necessarily increase the frequency of that clade over time. We compare the differences in clades including important co-variates such as protective HLA alleles and country of origin. Potential mediators of clade-associated differences in pathogenecity can only be resolved by further studies, particularly those that focus on the earliest events following HIV-1 infection.
